# The suspensor as a model system to study the mechanism of cell fate specification during early embryogenesis

**DOI:** 10.1007/s00497-018-0326-5

**Published:** 2018-02-23

**Authors:** Xiongbo Peng, Meng-Xiang Sun

**Affiliations:** State Key Laboratory of Hybrid Rice, College of Life Science, Wuhan University, Wuhan, 430072 China

**Keywords:** Suspensor, Zygote, Asymmetric division, Cell fate specification, Programmed cell death

## Abstract

***Key message*:**

**The advances in the suspensor**.

**Abstract:**

During early embryogenesis, the proembryo consists of two domains, the embryo proper and the suspensor. Unlike the embryo proper, which has been investigated extensively, research on the suspensor has been limited in past decades. Recent studies have revealed that the suspensor plays an important role in early embryogenesis and the process of suspensor formation and degeneration may provide a unique model for studies on cell division pattern, cell fate determination, and cell death. In this review, we briefly summarize the advances in research on the suspensor, which provide new insight in our understanding of the mechanism of early embryogenesis and show great potential for a unique model for future investigations.

## Introduction

Plant embryo development begins from the zygote, the fusion product of a sperm and egg cell. The zygote undergoes asymmetric cell division to generate a smaller apical cell and a larger basal cell. These two daughter cells establish cell lineages with different developmental fates (Menke and Scheres 2009). The small apical cell divides several times to form the embryo proper, while the larger basal cell divides a limited number of times, primarily to form the suspensor (Zhang and Laux 2011). In *Nicotiana tabacum*, the larger basal cell divides twice vertically to produce a four-cell suspensor (Zhao et al. 2013), while in *Arabidopsis thaliana*, the suspensor consists of 7–9 cells (Colette et al. 2015). The uppermost suspensor cell in eudicots differentiates into the hypophysis and eventually becomes part of the primary root meristem, while the remaining part of the suspensor will degenerate via programmed cell death (PCD) in the later stages of embryogenesis (Zhang and Laux 2011).

In comparison with studies on the embryo proper (Colette et al. 2015), research on the plant suspensor is—despite recent advances—still in its infancy. Although the suspensor is a transient structure of the proembryo, its generation, development, and degeneration processes involve important issues of developmental biology, such as cell polarity establishment, asymmetric cell division, cell fate specification, cell–cell communication, and PCD. In particular, its simple structure, typical morphology, limited cell number, and short-lived developmental process facilitate the monitoring of cell division and cell fate transition. These advantages enable the use of the suspensor or basal cell lineage as a model system to study the mechanism regulating those issues.

Many basic questions concerning the development of the suspensor remain unanswered. Since the first suspensor cell, the basal cell, is derived from the asymmetric zygote division, the impact of zygote division on basal cell fate specification, the status of basal cell specification in its developmental fate, and any distinct transcripts between the basal and apical cell, remain to be fully understood. Additionally, the suspensor is traditionally believed to be a supporting structure during plant embryo development, which pushes the embryo proper into the endosperm cavity and connects it to the surrounding maternal tissues and endosperm to facilitate the transfer of nutrients and plant hormones. Thus, it is naturally connected with maternal tissue, the embryo proper, and the endosperm in its developmental microenvironment. Whether any interaction exists between the suspensor and its environment, and how the environmental factors may influence suspensor developmental fate deserve investigation. Finally, the suspensor will degenerate, via PCD, in the later stages of embryogenesis. How PCD is regulated, and how this process may influence embryonic development are also among the interesting questions awaiting clear answers.

In this review, we introduce some advances in the study of suspensor development, primarily based on works from China over the last 10 years. Important results from others are also discussed. See Table 1 for an overview of suspensor development factors.

**Table 1:** Genes involved in zygote asymmetric division and suspensor development

Gene name	Organism	Function	Reference
*YDA*	*Arabidopsis*	Zygote elongation, asymmetric division, and suspensor formation	Lukowitz et al. (2004)
*GRD*	*Arabidopsis*	Zygote elongation and asymmetric division	Jeong et al. (2011)
*MPK3/6*	*Arabidopsis*	Zygote elongation and asymmetric division	Bayer et al. (2009)
*SSP*	*Arabidopsis*	Zygote elongation and asymmetric division	Bayer et al. (2009)
*ESF1*	*Arabidopsis*	Suspensor formation	Costa et al. (2014)
*WRKY2*	*Arabidopsis*	Zygote elongation and asymmetric division	Ueda et al. (2011)
*WOX8/9*	*Arabidopsis*	Apical–basal axis formation	Breuninger et al. (2008)
*HDG11/12*	*Arabidopsis*	Zygote elongation and asymmetric division	Ueda et al. (2017)
*GNOM*	*Arabidopsis*	Zygote elongation and asymmetric division	Mayer et al. (1993)
*ZAR1*	*Arabidopsis*	Zygote asymmetric division	Yu et al. (2016)
*RPL18aB*	*Arabidopsis*	Maintenance of suspensor identity	Xie et al. (2018)
*NtDRP*	*Nicotiana tabacum*	Orientation of the zygotic division plane	Zhao et al. (2016)
*KOD*	*Arabidopsis*	Triggering suspensor PCD	Blanvillain et al. (2011)
*NtCYS*	*Nicotiana tabacum*	Suppressing suspensor PCD	Zhao et al. (2013)
*NtCP14*	*Nicotiana tabacum*	Triggering suspensor PCD	Zhao et al. (2013)

## Transcript profiling analysis of the basal cell

In most higher plants, zygotes undergo asymmetric cell division to generate two daughter cells. The mechanisms underlying asymmetric cell division have been studied in organisms ranging from bacteria, yeast, worms, and flies to mammals (Li 2013). Asymmetric cell division can generate cellular diversity by differentially segregating RNA and protein determinants into the two daughter cells (Knoblich 2010).

To explain how apical and basal cells possess distinct cell fates in plant embryos, it has been proposed that the two cells contain different developmental determinants (Weterings et al. 2001). Researchers have identified several genes that are expressed differently in progeny after zygote division, such as DORNRÖSCHEN (DRN) (Cole et al. 2009), MERISTEM LAYER 1 (AtML1) (Lu et al. 1996), WUSCHEL HOMEOBOX2 (WOX2), and WOX8 (Haecker et al. 2004). These data suggest that the two daughter cells contain different transcripts that may be involved in cell fate specification.

To identify candidate developmental determinants for the fate of apical and basal cells in the whole genome, zygotes, two-celled proembryos, and individual basal or apical cells were collected to construct cDNA libraries (Ma et al. 2011; Zhao et al. 2011). Transcriptional profile analyses were performed to show how many different transcripts existed in these cells. Although the data showed that the transcript profile changed dramatically from zygotes to two-celled proembryos, the two-celled proembryos shared 33.5% expressed sequence tags (ESTs) with the zygotes, indicating that a great proportion of the transcripts in two-celled proembryos were derived from zygotes (Zhao et al. 2011). The results also showed that 16.0% of the apical cell EST clusters were present in basal cells and that 16.2% of the basal cell EST clusters were present in apical cells. Unexpectedly, only 19% of the ESTs showed significant expression level differences between apical and basal cells. Among approximately 200 genes, only three were found specifically expressed in the basal or apical cell (Ma et al. 2011). This indicates that although some of the zygote transcripts could be proportioned into the apical or basal cells following asymmetric zygote division, the majority of the transcripts were similar. This raised the possibility that the expression of a few cell-type specific or cell-type preferential genes, rather than distinct transcriptomes, triggered different developmental programs for the different cell fates.

## Basal cell fate in relation to zygotic elongation and asymmetric division

The asymmetric zygote division is a well-conserved and fundamental feature of embryogenesis in most angiosperms. Successful asymmetric zygote division requires two necessary processes, zygote elongation and accurate control of both position and orientation of the cell division plane.

Recent studies identified several factors that are required for zygote elongation and asymmetric division. In Arabidopsis, a signal pathway involved in zygote elongation was uncovered. YODA (YDA), encoding a mitogen-activated protein kinase kinase (MAPKK) kinase, and its downstream MAPKs MPK3/6 are required for zygote elongation and asymmetric division (Bayer et al. 2009; Lukowitz et al. 2004; Wang et al. 2007). Activation of YDA/MPK signaling in the zygote requires the membrane-associated receptor-like kinase SHORT SUSPENSOR (SSP). Surprisingly, SSP mRNA is transferred from the sperm cell to the egg and is then translated in the zygote (Bayer et al. 2009). Activated MPK3/6 directly phosphorylates a transcription factor, WRKY DNA binding protein 2 (WRKY2), which, in turn, leads to the upregulation of WOX8 transcription in the zygote to activate zygote elongation and asymmetric division (Ueda et al. 2011, 2017). Like WRKY2, maternally derived homeodomain GLABROUS (HDG) 11/12 directly binds to the WOX8 intron and activates its transcription (Ueda et al. 2017). Additionally, GNOM, a GDP/GTP exchange factor for small G-proteins of the ADP ribosylation factors (ARF) class and a predicted exo-polygalacturonase gene NIMNA are involved in zygote elongation and asymmetric division (Babu et al. 2013; Mayer et al. 1993). In mutants of these genes, the zygotes showed inhibited elongation and symmetric zygote division, resulting in the basal cell of mutant embryos failing to develop a normal suspensor. However, it is hard to determine whether these defects are due to the failure of zygote symmetrical division or its elongation because the two events are always coupled.

The recent research on zygote arrest 1 (ZAR1), a member of the receptor-like kinases (RLK)/Pelle family in Arabidopsis, has been useful to determine the contribution of zygote asymmetric division and its elongation to its daughter cell fate determination (Yu et al. 2016). In *zar1*, zygote elongation is normal, but zygote asymmetric division is impaired. Accordingly, both apical and basal cell fates were impaired, suggesting that the symmetric division itself is likely involved in daughter cell fate determination. ZAR1 kinase is activated by its binding with calmodulin and the heterotrimeric G protein Gβ subunit AGB1. The phenotype and expression pattern of cell linage-specific markers such as WOX2 and WOX8 in *agb1*-*2* is very similar to that in *zar1*-*2*. It suggests that ZAR1 and AGB1 may function through their interaction in the asymmetric division of zygote and specification of apical and basal cell lineages. These data suggest that ZAR1 might act as an integrator for intracellular Ca^2+^ and heterotrimeric G protein signaling with extracellular signals during zygote development and cell fate determination (Yu et al. 2016).

The above-mentioned genes deal with the control of the position of the zygotic division plane, that is, control of symmetric or asymmetric zygote division. However, the factors responsible for the orientation of the zygotic division plane remain to be determined. It has been shown that microtubules regulate zygote cell elongation, whereas actin filaments promote polar migration of the nucleus to the apical tip (Kimata et al. 2016). Although the cytoskeleton plays an important role in zygotic elongation and asymmetric division, its effect on basal cell fate needs further investigation. A recent study showed that NtDRP, which encodes a dynamin-related protein, plays an important role in maintaining the orientation of the zygotic division plane in tobacco (Zhao et al. 2016). NtDRP is expressed in the zygote, apical and basal lineages, and early embryos, but not in sperm or egg cells (Ning et al. 2006; Zhao et al. 2011, 2016). NtDRP modulates microtubule spatial organization and spindle orientation during early embryogenesis. Downregulation of *NtDRP* caused an incorrect orientation of the zygotic division plane, resulting in the formation of an embryo-like structure without a typical suspensor. This observation suggests that NtDRP-dependent zygotic division plane orientation is essential for the differentiation of both apical and basal cell lineages, especially for suspensor formation (Zhao et al. 2016). In conclusion, asymmetrical cell division of the zygote appears important for basal cell fate and the establishment of the suspensor, as well as for embryonic pattern formation.

## Developmental fate of the basal cell lineage in relation to the apical cell lineage

The suspensor functionally transports nutrients and auxins from maternal tissue to the embryo proper and thus is structurally connected with both maternal tissue and the embryo proper, which are the major microenvironmental factors important for suspensor development. Whether these factors play any role in suspensor cell fate specification and development is of great interest. Specifically, how the basal cell lineage may interact with the apical cell lineage has long drawn researchers’ attention. In some mutants, the embryo proper is abnormal and the suspensor cells can develop into proembryos or globular embryo-like structures (Marsden and Meinke 1985; Sanmartín et al. 2011; Schwartz et al. 1994; Yadegari et al. 1994; Zhang and Somerville 1997). Whether these genetic mutations have direct effects on suspensor cells remains unknown. It has been proposed that cells of a basal cell lineage have embryogenic potential that is suppressed by the embryo, and a basal cell lineage may develop into an embryo if relieved from the suppression of the embryo proper (Schwartz et al. 1994; Vernon and Meinke 1994; Zhang and Somerville 1997).

Recently, direct evidence has supported the hypothesis by use of an in vivo living cell laser ablation system (Gooh et al. 2015; Liu et al. 2015). Liu et al. showed that, after the connection between the embryo proper and the suspensor is removed by a laser, the newly formed top suspensor cell can develop into a second embryo with the same morphology and expression of embryo-specific genes. Suspensors at stages before the formation of the globular embryo have embryogenic potential, while suspensors at stages after the heart embryo stage no longer have embryogenic potential, indicating that suspensor cells possess embryogenic potential only at some early stages (Liu et al. 2015). Gooh et al. showed that the basal cell lineage possessed embryogenic potential even at the two-celled proembryo stage. After laser ablation of the apical cell, the basal cell first divided horizontally. The upper daughter cell then divided vertically, comparable to division of the apical cell. The apical-like basal cell divided similarly to give rise to a 16-celled embryo (Gooh et al. 2015). These observations confirmed that the embryogenic potential was suppressed by the embryo proper because the second embryo, generated from the suspensor, occurred only after removing the embryo proper.

Liu et al. (2015) further revealed that redistribution of auxin in the suspensor after ablation of the apical cell lineage likely resulted in the developmental fate transition of the new top suspensor cell. During normal embryogenesis, auxin polar transportation is from maternal tissue to the embryo via the suspensor before the globular embryo stage, and the suspensor usually contains a low level of auxin. The new top suspensor cell, however, accumulated a higher level of auxin after removal of the embryo proper. As a result, the new top cell of the suspensor started to divide and ultimately developed into a second embryo. Additionally, when laser-ablated suspensors were isolated and cultured in medium with the auxin transport inhibitor *N*-1-naphthylphthalamic acid (NPA), the suspensor cells failed to generate a second embryo (Liu et al. 2015). These lines of evidence indicate that proper levels of plant hormones, and their polar transport from maternal tissue to the suspensor cell, are both key to the cell fate transition of suspensor cells in triggering embryogenesis.

A further question is raised: how do the suspensor cells maintain their cell identity during the normal embryogenesis to ensure the nutrients and auxin transportation for early embryo proper development? A recently study showed that RPL18aB, a ribosomal protein, is required for the maintenance of suspensor identity (Xie et al. 2018). The mutation of *RPL18aB* resulted in its suspensor cells developing into an embryo-like structure. The suspensor of *rpl18aB* expressed embryo proper marker DRN and lost the expression of suspensor marker WOX8. Suspensor-specific expression of *RPL18aB* was sufficient to rescue the cell proliferation defects of *rpl18aB* suspensor. These results provide a valuable clue to seek how the suspensor cells maintain its identity during early embryogenesis.

## Start point of basal cell fate specification

After zygote division, the basal and apical cell show distinct morphologies and transcript differences, suggesting their cell fate has been specified. As described previously, embryonic cell fate specification in animals has three types: autonomous cell fate specification, conditional specification, and syncytial specification (Davidson 1990). However, detailed investigations of the cell fate specification type involved in plant embryonic development have yet to be carried out. Whether the cell fate has been specified after zygote division and what type of cell specification occurs during suspensor formation are questions that remain to be answered.

It seems likely that cell fate specification of the basal cell lineage is autonomous, because the basal and the apical cells show different transcripts and development fates after the first asymmetric zygote division. However, two groups have confirmed that the basal cell lineage is capable of a cell fate transition to the embryo when the apical cell lineage was removed by laser ablation (Gooh et al. 2015; Liu et al. 2015), indicating that the cell fate of the basal cell lineage was not specified. Notably, the basal cell lineage showed embryonic potential when still attached to maternal tissue during its development in the embryo sac. To determine basal cell fate specification, the influence of maternal tissue should be evaluated. Recently, Qu et al. (2017) evaluated the role of maternal tissue and the apical cell lineage in basal cell fate specification using an in vitro system. They found that a single basal cell could develop into a typical suspensor and retain the same cell division pattern and cell identity, indicating that basal cell fate is an autonomous specification process and does not depend on interactions with the apical cell or maternal tissue. The isolated apical cells could finally develop into small globular embryos in vitro. However, a new suspensor was not regenerated from the apical cell lineage, suggesting that none of the cells from the apical cell lineage had the ability to generate a second basal cell lineage (Qu et al. 2017).

It was concluded that the initial round of cell fate specification of the suspensor cell was autonomous and had already occurred at the two-celled proembryo stage. Although suspensor cell fate transition is still possible thereafter, the presentation of embryonic potential of suspensor cells is a conditional process that depends on developmental cues from both the apical cell lineage and maternal tissue.

## Suspensor degeneration: a molecular switch for triggering PCD

The suspensor is only required in the early stages of embryogenesis, after which it subsequently degenerates. Although it has been known that the suspensor is a temporary organ for a century, the molecular mechanism triggering its degeneration has remained largely unknown. In Arabidopsis, a suspensor-expressed peptide, KISS OF DEATH (KOD), has been shown to positively regulate PCD (Blanvillain et al. 2011). A loss-of-function mutation resulted in a delay of suspensor PCD, and overexpression caused ectopic cell death in seedlings. Although it was shown that KOD expression was sufficient to cause death in leaves or seedlings and to activate caspase-like activities, the mechanism of KOD regulation of suspensor PCD is unknown.

Recently, two proteins have been found that antagonistically balance each other in the control of suspensor PCD (Zhao et al. 2013). In tobacco, suspensor PCD starts from the 32-cell stage, with the death signal progressing gradually from the basal cell to the uppermost cell of the suspensor. The NtCYS protein, a member of the cystatin family, is restricted to the basal cell of the suspensors, where PCD begins. Decreasing the expression of *NtCYS* resulted in premature death of the basal cells, while overexpressing *NtCYS* delayed basal cell death (Zhao et al. 2013). These findings indicated that NtCYS acts to suppress cell death in the basal cell lineage.

As a cystatin, *NtCYS* was suggested to inhibit enzymes known as cysteine proteases. In fact, *NtCYS* RNA interference plants showed increased cathepsin H-like protease activity in two-celled proembryos. Twenty genes encoding cysteine proteases were found to be expressed in two-celled proembryos. Among them, only one cathepsin H-like protease, NtCP14, interacted with *NtCYS.* Overexpressing *NtCP14* triggered precocious cell death in the basal cell lineage, while silencing *NtCP14* delayed cell death in the suspensor. This work determined that NtCYS-NtCP14 acts a bipartite molecular module to control PCD in the suspensor: NtCYS inhibits NtCP14 activity during early suspensor development, but when the concentration of NtCYS is decreased, NtCP14 activity increases and initiates PCD (Zhao et al. 2013).

## Influence of the suspensor on development of the embryo proper

The suspensor is believed to be key in embryo development with its function of transferring nutrients and phytohormones to the embryo (Kawashima and Goldberg 2010). Little is known about its role in early embryogenesis or in later embryo pattern formation. The first evidence came from the study of suspensor PCD in tobacco (Zhao et al. 2013). Decreasing the expression of NtCYS caused precocious PCD in the basal domain of the proembryo, further resulting in embryos arrested before the eight-cell stage. Cessation of embryogenesis was correlated with aberrant patterns of cell division in the apical cell lineage.

The in vivo embryo laser ablation systems provided additional data supporting the role of the suspensor in the development of the embryo (Gooh et al. 2015; Liu et al. 2015). After removing the basal cell of a two-celled proembryo, the apical cell divided to form a small globular embryo at the 4- to 32-cell stage. However, aberrant cell division in the embryo was observed, with an 11- to 16-h delay in the duration of cell division compared with that of the control embryo (Gooh et al. 2015). The embryonic behavior at three stages of development following laser ablation of the suspensor was observed (Liu et al. 2015). After breaking the connection between the embryo proper and the suspensor of eight-celled embryos, only 3.51% of the embryos developed into heart embryo-like structures. The majority of the embryos still arrested at the globular embryo stage. After breaking the connection in 32-cell embryos, 57.81% of the embryos developed into heart-stage embryos. After cutting off the suspensor, 90.91% of the heart-stage embryos developed into torpedo embryos. Development of the 8- and 32-cell embryos without suspensors was slower than that of embryos with suspensors, especially for the eight-celled embryos. However, the developmental speeds of the heart-stage embryos with or without suspensors were similar. These results show that the suspensor is important for the early development of embryos, in a stage-dependent manner. Moreover, even at the 32-cell embryo stage, if the original hypophysis cell derived from the top cell of the suspensor was removed, the development of the globular embryo into a transition stage was severely impaired (Liu et al. 2015).

## Summary and perspective

Available data indicate that basal cell lineage or suspensor development can be a valuable system for the investigation of mechanisms regulating cell division pattern, cell fate specifications, and the relationship between them. It also provides a unique chance to investigate the role of cell–cell interactions in cell fate determination. To date, it is known that asymmetric zygote division plays a key role in basal cell fate determination. At least, at the two-celled proembryo stage, the basal cell has already been specified toward suspensor cell fate. However, its subsequent cell fate specification is directly controlled by both maternal tissue and the apical cell lineage. The suspensor cells still possess embryogenic potential, which is normally suppressed by the apical cell lineage, as long as it is connected to the maternal tissue. Auxin polar transport from maternal tissue to the suspensor plays an important role in suspensor cell fate transition to embryo cells. Finally, a basal cell-specific molecular mechanism controls the timely degeneration of the suspensor cells. Predegeneration of the suspensor will disturb early embryo development, indicating its essential role in early embryogenesis. These findings open a new window to study in detail the mechanism(s) underlying suspensor generation, development, and degeneration.

Our understanding of suspensor development has increased greatly over the past 10 years; however, many key issues still remain to be determined. Although research has confirmed transcript differences between apical and basal cells, the mechanisms controlling the polar distributions of specific transcripts in the zygote and the proportions of these specific transcripts into the apical or basal cell are unknown. Some genes have been found to contribute to the asymmetric division of the zygote and show specific localization in apical or basal cells, while the molecules controlling the fate of the daughter cells of the zygote need further investigation. Cell division is always horizontal in the suspensor, unlike in the embryo proper (Zhao et al. 2017). This division pattern in the suspensor is likely beneficial to auxin polar transport to the embryo proper, because PIN-FORMED 7 (PIN7) is localized to the apical sides of suspensor cells before the eight-cell embryo (Friml et al. 2003). However, what controls the cell division pattern in the suspensor and how this division pattern may contribute to auxin transportation are unknown. Although NtCYS activity is known to control PCD in the suspensor (Zhao et al. 2013), it is still unclear what signal controls the spatiotemporal expression of NtCYS, and where the signal comes from in an ovule. Additionally, the basal cell divides only a limited number of times to form the suspensor. What controls the cell cycle of suspensor cells deserves careful study.

Further transcriptome analysis based on new sequencing techniques will identify additional genes specifically expressed in apical and basal cells, the hypophysis, and the suspensor, which will help in elucidating the cell fate determinants of these cells. Additionally, investigating the signal cascades of the known genes involved in suspensor development will deepen our understanding of the mechanisms of this process.

### Author contribution statement

Mengxiang Sun and Xiongbo Peng wrote and reviewed the manuscript.
